# Metagenomic data of the microbial community of the chemocline layer of the meromictic subarctic Lake Bolshie Hruslomeny, North European Russia

**DOI:** 10.1016/j.dib.2019.103800

**Published:** 2019-03-02

**Authors:** Vitaly V. Kadnikov, Alexander S. Savvichev, Andrey V. Mardanov, Alexey V. Beletsky, Nikolai V. Ravin, Nikolai V. Pimenov

**Affiliations:** a Institute of Bioengineering, Research Center of Biotechnology of the Russian Academy of Sciences, Moscow, Russia; b Winogradsky Institute of Microbiology, Research Center of Biotechnology of the Russian Academy of Sciences, Moscow, Russia; c Faculty of Biology, Lomonosov Moscow State University, Moscow, Russia

## Abstract

The Lake Bolshie Hruslomeny is located on the shores of the Kandalaksha Bay of the White Sea, North European Russia. This lake, formed from the sea bay and still retaining the subsurface connection with the sea, is meromictic, with a fresh oxygenated upper layer and an anoxic brackish hypolimnion with high concentrations of methane and hydrogen sulphide. To characterize the microbial communities involved in the carbon and sulfur cycles in the lake, we sequenced the metagenome of a water sample collected at the chemocline level. At the phylum level, *Chlorobi*, *Proteobacteria*, *Bacteroidetes* and *Firmicutes* were the most numerous groups. The obtained data will help investigate the diversity and ecological role of the microbial community in the Lake Bolshie Hruslomeny and provide insight into the biogeochemical processes in subarctic lakes. The raw sequencing data is available from the NCBI Sequence Read Archive (SRA) database under the BioProject PRJNA503531.

**Table:** Specifications table

Subject area	Biology
More specific subject area	Metagenomics
Type of data	Metagenomic sequences
How data was acquired	Shotgun DNA sequencing using Illumina HiSeq 2500
Data format	Raw and analyzed
Experimental factors	Environmental sample
Experimental features	The water sample from the chemocline level (at 4.25 m depth) of the Lake Bolshie Hruslomeny was collected and then the total DNA was extracted and sequenced.
Data source location	Lake Bolshie Hruslomeny in the Murmansk region of Russia (66.716380 N, 32.858134 E)
Data accessibility	Data is submitted to NCBI with BioProject PRJNA503531 and it is in the public repository. The direct URL to data is https://trace.ncbi.nlm.nih.gov/Traces/sra/sra.cgi?run=SRR8146599
Related research article	None

**Table:** 

**Value of the data** • The obtained data provided an insight of the composition of the microbial community of the chemocline of the meromictic subarctic Lake Bolshie Hruslomeny. • The analysis revealed the prevalence of anoxygenic phototrophic bacteria of the phylum Chlorobi in the chemocline. • This metagenome is valuable for the study of microbial processes related to methane and sulfur cycles in Lake Bolshie Hruslomeny. • The raw metagenome data is publicly available for further analysis and comparison of metagenomes among various meromictic lakes.

## 1 Data

Meromictic lakes are the subject of research in the field of limnology, biogeochemistry and microbiology [1], [2]. Bolshie Hruslomeny Lake is located on the shores of the Kandalaksha Bay of the White Sea, North European Russia. This lake was originally a sea gulf, artificially separated from the sea by a dam at the beginning of the 20th century. The maximum depth of the lake is about 18 m. The upper epilimnion layer (0–3.5 m) is fresh and oxygenated. The water in the hypolimnion (below 5 m) is anoxic, brackish (up to 22‰) due to infiltration of seawater, has high concentrations of methane (up to 1.8 mM) and hydrogen sulphide (up to 17.8 mM). The highest rates of methane oxidation and anoxygenic photosynthesis were observed in the chemocline zone. This lake is an interesting object for studying the microbial processes related to the methane and sulfur cycles. To characterize the microbial communities involved in these processes, we performed a metagenomic shotgun sequencing of a water sample collected at the chemocline level.

**Fig. 1:**
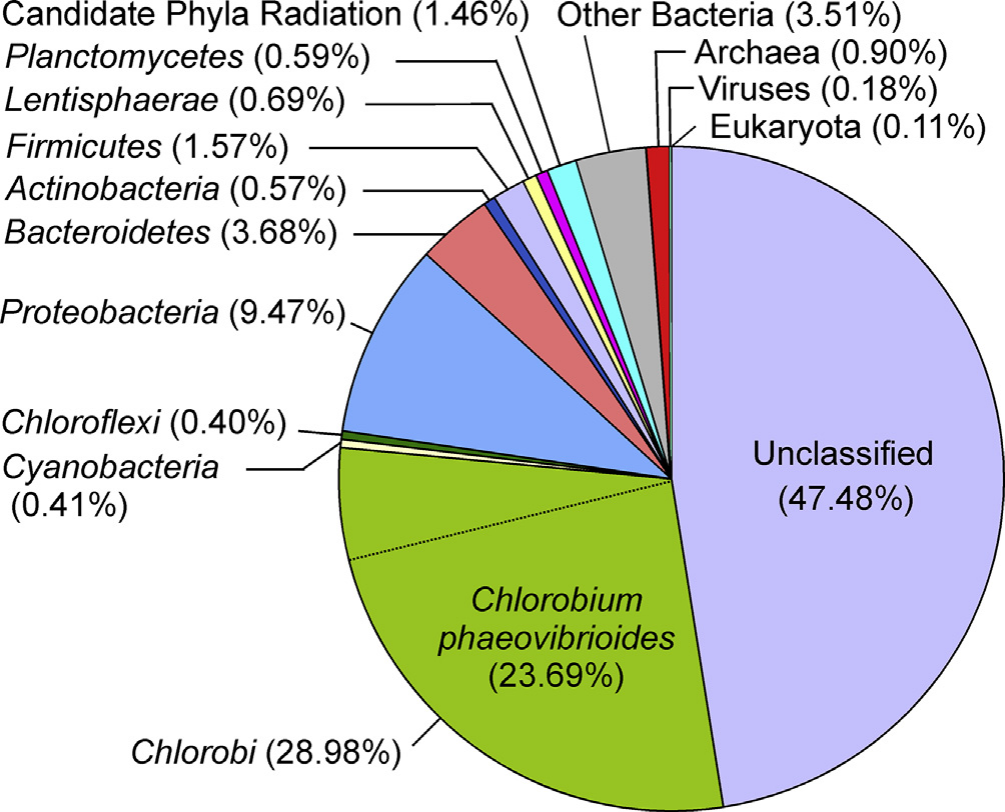
Taxonomic assignment of metagenome of the chemocline of the Lake Bolshie Hruslomeny.

Of the 10,845,687 sequencing reads, 51.33% were assigned to Bacteria, 0.90% to Archaea, 0.11% to Eukaryota, 0.18% to viruses, while other reads were not classified. At the phylum level, *Chlorobi* (28.98%), *Proteobacteria* (9.47%, mostly members of the class delta), *Bacteroidetes* (3.68%), and *Firmicutes* (1.57%) were the most abundant, as shown in Fig. 1. At the species level, a single bacterium, *Chlorobium phaeovibrioides*, accounted for 23.69% of all reads. The obtained data will help investigate the diversity and ecological role of the microbial community in the Lake Bolshie Hruslomeny and provide insight into the various biogeochemical processes in subarctic lakes.

## 2 Experimental design, materials, and methods

### 2.1 Sample collection and preparation

The water sample was collected from the chemocline level (at a depth of 4.25 m) of the Lake Bolshie Hruslomeny in March 2017 using sterile plastic containers. The concentration of microorganisms, according to microscopic estimation, was about 1.9 ×10^7^ cells/ml. The sample was delivered to the laboratory on ice for 3 h. Cells from 75 ml of water were collected on 0.22 μm cellulose nitrate membranes (Sartorius, Germany) using a Sartorius filtration unit.

### 2.2 DNA extraction

The filters were frozen in liquid nitrogen and then ground and mixed with TE buffer (pH 8.0) in a 37 °C water bath. Total DNA was extracted using Power Soil DNA Isolation Kit (MO BIO Laboratories Inc, Carlsbad, USA). The quality and concentration of the extracted DNA sample was measured using Qubit® dsDNA HS Assay Kit (Life Technologies), followed by agarose gel electrophoresis. About 1 μg of total DNA (20 ng/μL) was isolated.

### 2.3 Sequencing and taxonomic analysis

Sequencing library was prepared using 100 ng of DNA with the Nextera DNA Library preparation kit (Illumina Inc., USA) following the manufacturer's instructions. The sequencing of this library on the Illumina HiSeq2500 platform using HiSeq Rapid Run v2 sequencing reagents generated 11,061,025 single-end reads with a length of 250 nt (2.8 Gbp in total). Primer and quality trimming were performed with Cutadapt v. 1.17 [3] and Sickle v. 1.33 (https://github.com/najoshi/sickle), respectively. Cutadapt was used with the default settings, and Q30 score was used for the Sickle. 10,845,687 reads with an average length of 193 nt were retained after processing.

Taxonomic classification of filtered reads was carried out using the Kaiju program [4], with default parameters. A microbial subset of the NCBI non-redundant protein database, also including fungi and microbial eukaryotes (nr + euk option), was used as a reference database. 5,695,944 of 10,845,687 reads were classified.
